# Tropism of mesenchymal stem cell toward CD133^+^ stem cell of glioblastoma in vitro and promote tumor proliferation in vivo

**DOI:** 10.1186/s13287-018-1049-0

**Published:** 2018-11-09

**Authors:** Lorena Favaro Pavon, Tatiana Tais Sibov, Andrea Vieira de Souza, Edgar Ferreira da Cruz, Suzana M. F. Malheiros, Francisco Romero Cabral, Jean Gabriel de Souza, Pamela Boufleur, Daniela Mara de Oliveira, Silvia Regina Caminada de Toledo, Luciana C. Marti, Jackeline Moraes Malheiros, Fernando F. Paiva, Alberto Tannús, Sérgio Mascarenhas de Oliveira, Ana Marisa Chudzinski-Tavassi, Manoel A. de Paiva Neto, Sérgio Cavalheiro

**Affiliations:** 1 Department of Neurosurgery, Federal University of São Paulo, São Paulo, Brazil; 2 Experimental Research Center, Hospital Israelita Albert Einstein, São Paulo, Brazil; 3 Discipline of Nephrology, Federal University of São Paulo, São Paulo, Brazil; 4 Laboratory of Molecular Biology, Butantan Institute, São Paulo, Brazil; 5 Centre of Excellence in New Target Discovery (CENTD), Butantan Institute, São Paulo, Brazil; 6 Department of Genetics and Morphology, University of Brasília, Brasília, Brazil; 7 Pediatric Oncology Institute, Grupo de Apoio ao Adolescente e à Criança com Câncer (GRAACC), Federal University of São Paulo, São Paulo, Brazil; 8 São Carlos Institute of Physics, São Paulo University, São Carlos, Brazil; 9 Laboratory of Cellular and Molecular Neurosurgery, Federal University of São Paulo, Rua Napoleão de Barros, n. 626 –Vila Clementino, São Paulo, SP 04024-002 Brazil

**Keywords:** CD133^+^ cells, MSCs, Tropism, Chemokines, Experimental model, Exosomes

## Abstract

**Background:**

Previous studies have demonstrated remarkable tropism of mesenchymal stem cells (MSCs) toward malignant gliomas, making these cells a potential vehicle for delivery of therapeutic agents to disseminated glioblastoma (GBM) cells. However, the potential contribution of MSCs to tumor progression is a matter of concern. It has been suggested that CD133^+^ GBM stem cells secrete a variety of chemokines, including monocytes chemoattractant protein-1 (MCP-1/CCL2) and stromal cell-derived factor-1(SDF-1/CXCL12), which could act in this tropism. However, the role in the modulation of this tropism of the subpopulation of CD133^+^ cells, which initiate GBM and the mechanisms underlying the tropism of MSCs to CD133^+^ GBM cells and their effects on tumor development, remains poorly defined.

**Methods/results:**

We found that isolated and cultured MSCs (human umbilical cord blood MSCs) express CCR2 and CXCR4, the respective receptors for MCP-1/CCL2 and SDF-1/CXCL12, and demonstrated, in vitro, that MCP-1/CCL2 and SDF-1/CXC12, secreted by CD133^+^ GBM cells from primary cell cultures, induce the migration of MSCs. In addition, we confirmed that after in vivo GBM tumor establishment, by stereotaxic implantation of the CD133^+^ GBM cells labeled with Qdots (705 nm), MSCs labeled with multimodal iron oxide nanoparticles (MION) conjugated to rhodamine-B (Rh-B) (MION-Rh), infused by caudal vein, were able to cross the blood-brain barrier of the animal and migrate to the tumor region. Evaluation GBM tumors histology showed that groups that received MSC demonstrated tumor development, glial invasiveness, and detection of a high number of cycling cells.

**Conclusions:**

Therefore, in this study, we validated the chemotactic effect of MCP-1/CCL2 and SDF-1/CXCL12 in mediating the migration of MSCs toward CD133^+^ GBM cells. However, we observed that, after infiltrating the tumor, MSCs promote tumor growth in vivo probably by release of exosomes. Thus, the use of these cells as a therapeutic carrier strategy to target GBM cells must be approached with caution.

## Background

Glioblastoma (GBM) is the most common central nervous system (CNS) malignancy, with very limited therapeutic options due to its infiltrative nature and high resistance to radiation therapy and chemotherapy [1–3]. These characteristics could be justified by the competence of the tumor cells to stem cell lines. A possible hypothesis about tumor stem cells describes that tumors are maintained for a fraction of rare cells having stem cell properties, and the nature defined by the formation of tumor neurospheres, which contain a subpopulation of CD133^+^ cells that initiate gliomas [4–6]. The remainder stems from previously unknown CD133^−^ tumor cells with apparent stem cell-like properties but distinct molecular profiles and growth characteristics both in vitro and in vivo [7].

Despite recent therapeutic advances, the outcome of GBM remains dismal. Some studies have successfully demonstrated that mesenchymal stem cells (MSCs) have a strong tropism for glioma and may act as a vehicle for drug delivery [8–10], or even may exert immunoregulatory activity, representing an attractive therapeutic strategy for residual neoplastic foci inconventional therapy [11–16]. Other studies, however, suggest that MSCs may contribute to tumor growth, or that the multipotent and immunomodulatory properties of these cells can create conditions for tumor development, progression and even metastatic spread [17–19]. This communication, MSCs and tumor cell, possibly occur through exosomes secreted by MSCs [20, 21]. Exosomes are microvesicles formed by endosomal membrane invagination, that later fuse to the plasmatic membrane and are released out of the cell [22]. Exosomes have an evolutionary conserved set of proteins including tetraspanins (CD63 and CD9) [23]. Increasing evidence has suggested that exosomes have significant roles in tumor growth, progression, metastasis, and drug resistance [24]. However, the true role of MSC-derived exosomes in the maintenance and propagation of gliomas is unclear.

Therefore, a better understanding of the molecular events that govern MSC homing and intercellular communication is crucial for the development of a clinically applicable tumor targeting strategy.

Certain chemokines and growth factors, including vascular endothelial growth factor (VEGF), interleukin-8 (IL-8), transforming growth factor-β (TGF-β), and neurotrophin-3 (NT-3) released from mature glioma cells, have been reported to mediate the tropism of MSCs for gliomas [25–27]. In addition, several other chemokines are secreted by glioma cells, including monocytes chemoattractant protein-1 (MCP-1/CCL2) and stromal cell-derived factor-1(SDF-1/CXCL12) [28–30].

For these reasons, we investigated the role of CD133^+^ glioma stem cell, defined by the formation of GBM neurospheres, aiming to narrow down a possible chemotactic relationship with MSCs, through research into specific binding of MCP-1/CCL2 and SDF-1/CXCL12 in CD133^+^ cells, considering the presence of their receptor CCR2/CXCR4 in MSCs. Our work also aims to (i) establish in vivo assays to evaluate the tumorigenicity of CD133^+^ cells in conjunction to with the migration of MSCs toward GBM, (ii) assess MSCs contribution to tumor development, invasion and metastatic dissemination, and (iii) the role of exosomes release by MSCs in these processes.

## Methods

In this study, we analyzed five samples of human primary GBM obtained from adult patients undergoing resection at the Department of Neurosurgery, Federal University of São Paulo, São Paulo, Brazil. All patients gave signed, informed consent for their tissues to be used for scientific research. The pathologist according to the World Health Organization classification criteria (WHO 2016), using molecular parameters in addition to histology by Louis et al. [31] evaluated the tumors.

### Establishment of the GBM primary cell culture

Fresh GBM samples were washed and minced in phosphate-buffered saline (PBS) (1X); this was followed by enzymatic dissociation with collagenase-I 0.3% (Sigma-Aldrich). The isolated cells were resuspended in Dulbecco’s modified Eagle’s medium-low glucose (DMEM-LG; Gibco/Invitrogen Corporation) supplemented with 200 mM of l-glutamine, antibiotic–antimycotic (10,000 U/mL of sodium penicillin, 10,000 μg/mL of streptomycin sulfate, and 25 μg/mL of amphotericin B; Thermo Fisher Scientific), and 10% fetal bovine serum (Thermo Fisher Scientific). The cells were seeded in 25-cm^2^ culture flasks and maintained at 37 °C with 5% CO_2_. The experiments described in this work were performed with cells in the second or third cell passages.

### GBM-derived neurosphere culture

The tumor cells, obtained in the primary culture of five samples of GBM, described above were resuspended in *tumor brain stem cell medium* (TBSCM) (Dulbecco’s modified Eagle’s medium/F12; Thermo Fisher Scientific), supplemented with N-2 (Thermo Fisher Scientific), epidermal growth factor (EGF; 20 ng/mL; Thermo Fisher Scientific), basic fibroblast growth factor (bFGF; 20 ng/mL; Thermo Fisher Scientific), leukemia inhibitory factor (LIF; 10 ng/μl; EMD Millipore), and B-27(1:50; Thermo Fisher Scientific) by Lenkiewicz et al. [32]. Viable cells were seeded in 24-well plates at a density of 2 × 10^4^ cells/cm^2^. The cells were maintained in a humidified incubator (Thermo Fisher Scientific, Waltham, MA) with 5% CO^2^ at 37 *°*C. The experiment was reproducible in the five GBM samples.

### Purification of the GBM cells with CD133 microbeads and preparation of the tumor subspheres

The neurosphere colonies were dissociated using StemProAccutase (Thermo Fisher Scientific) and maintained at room temperature for 10 min. The cells were labeled with CD133 magnetic microbeads (MACS; Miltenyi Biotec) and selected with an affinity column (Miltenyi Biotec). To verify the separation efficiency, the CD133^+^ cells were stained with CD133/2PE and evaluated by using flow cytometry (FACSAria, BD Biosciences, San Jose, CA) and analyzed with FACSDiva software (BD Biosciences, San Jose, CA) [33, 34]. Subsphere formation was observed in only the CD133^+^ cells and was documented by using phase-contrast microscopy (Olympus IX51).

### Immunophenotyping of CD133^+^ GBM cells by using flow cytometry

Subspheres were harvested with StemProAccutase Cell Dissociation Reagent (Thermo Fisher Scientific, Carlsbad, CA) and washed with PBS (pH = 7.4). For intracellular staining, the cells were fixed (FACS Lysing Solution, BD Biosciences) and permeabilized (Permeabilization Solution 2, BD Biosciences, San Jose, CA). Human monoclonal antibody CD133/2 PE (clone: 133/2; Miltenyi Biotec, Bergisch Gladbach, Germany) (BD Biosciences, San Diego, CA) was used. The data were acquired with a FACSAria flow cytometer (BD Biosciences, San Jose, CA) and analyzed by using FACSDiva (BD Biosciences, San Jose, CA) or FlowJo software (Tree Star, Ashland, OR).

### Transmission electron microscopy (TEM) of GBM subspheres and CD133^+^ tumor cells

GBM subspheres and CD133^+^ cells were fixed in 1% glutaraldehyde and 0.2 M of cacodylate buffer for 2 h at 4 °C, according to previously described methods for TEM by Pavon and colleagues [34]. Semithin and ultrathin sections were obtained using a Porter Blum ultramicrotome. The ultrathin sections (70 nm) were placed on copper grids and stained with uranyl acetate and lead citrate. The grids were studied and photographed under a TEM (Philips CM100).

### CD133^+^ GBM cell labeling with Qdots (705 nm)

Approximately 10^3^ CD133^+^ GBM cells were plated in 24-well plates for approximately 24 h at 37 °C with 5% CO^2^. The cells were incubated, for 60 min, in Dulbecco’s modified Eagle’s medium (DMEM)/F12 with Qdots (705 nm), pre-mix: 1 μL A_Qtracker, and 1 μL B_Qtracker in 200 μL DMEM/F12. After incubation, DMEM/F12 was removed and cells were washed twice with PBS (1X). CD133^+^ cells were analyzed using a fluorescence microscope (IX51 Olympus, Tokyo, Japan) with emission filter fluorescence (705 nm) and excitation filter (405-665 nm) to detect the Qdots (705 nm). For the study of intracellular distribution of Qdots, CD133^+^ cells were fixed with 4% paraformaldehyde and the cell nuclei were labeled with diamidino-2-phenylindole (DAPI, Sigma-Aldrich) and analyzed with an IX51 fluorescence microscope (Olympus, Tokyo, Japan).

### Isolation and culture of umbilical cord-derived MSCs (UC-MSC)

Five umbilical cord samples were collected with the informed consent of the donor’s mother, with protocol approval from the ethics committee for research at Federal University of São Paulo, São Paulo, Brazil. The samples were processed and cultured for 21 days, according to previously described methods by Sibov and colleagues [36]. After 3 weeks, UC-MSCs with fibroblast morphology were the dominant cells in the culture. UC-MSCs were characterized immunophenotyping (CD29, CD44, CD73, CD90, CD105, CD166 markers) by flow cytometry and were differentiated into mesodermal lineages (adipogenic and osteogenic differentiation) according to established protocols [35, 36]. All experiments were performed with all five established cellular lineages in the fourth passage.

### In vitro MSCs labeling with multimodal iron oxide nanoparticles (MION) conjugated to rhodamine-B (Rh-B) (MION-Rh) and intracellular MION-Rh detection

Approximately 1 × 10^4^ MSCs were plated into 24-well plates. The cells were incubated overnight (approximately 18 h) in DMEM-LG with 40 μg Fe/mL MION-Rh at 37 °C and 5% CO_2._ After incubation, the culture medium was removed, and the cells were washed twice with PBS (1X) to remove residual extracellular MION-Rh. MSCs were treated with 0.25% TrypLE Express (Gibco/Invitrogen Corporation). Cells were immediately harvested, visualized, and manually counted using 0.4% Trypan Blue (Gibco/Invitrogen Corporation) under an inverted microscope (IX51 Olympus, Tokyo, Japan). MSCs were washed twice with PBS (1X) and fixed with 4% paraformaldehyde. The fixed cells were subsequently subjected to fluorescence analysis using diamidino-2-phenylindole (DAPI, Sigma-Aldrich) to label the cell nuclei and an Rh-B filter (530 nm and 550 nm) to detect the MION-Rh. All cells were analyzed using a fluorescence microscope (IX51 Olympus, Tokyo, Japan).

### RT-PCR analysis of MCP-1/CCL2, SDF-1/CXCL12 and CCR2, CXCR4 mRNA

Total RNA was extracted from CD133^+^ GBM cells and MSCs using TRIzol (Invitrogen, Carlsbad, CA) according to the manufacturer’s instructions. The RNAs were reverse transcribed using the SuperScript III First-Strand synthesis system (Invitrogen) with oligo (dT) as primers. PCR reactions were performed in a DNA Thermal Cycler 480 (PerkinElmer Life Sciences, Boston, MA), and the amplifications were carried out in a volume of 12.5 μl containing 1 μg cDNA, 10 mM Tris-HCl, 50mMKCl, 0.2 mM of each dNTP, 1.5 mM MgCl_2_, 10 pmol of each primer, and 0.1 U Taq polymerase, for 5 min at 94 °C for initial denaturing, followed by 32 cycles of 94 °C for 30 s, 60 °C for 30 s, 72 °C for 30 s, and a final incubation at 72 °C for 7 min. PCR products were sized fractioned by electrophoresis on 2% agarose gels and visualized with ethidium bromide. The specific primers used are shown in Table 1.

**Table 1:** Gene-specific primers for RT-PCR

Gene	Gene Bank accession no.	Oligonucleotide (5′-3′)
CCR2	NC_000003.12	Forward: GCC GCT GCT CAT CAT GGG T
		Reverse: TGC CTC TTC TTC TCG TTT CGA
CXCR4	NC_000002.12	Forward: GGG TGG GGT GGT GGT GAG TAT T
		Reverse: AGG GGG TTG GGG TTG TGG TG
MCP-1/CCL2	NC_000017.11	Forward: ATG CAA TCA ATG CCC CAG TC
		Reverse: TGC AGA TTC TTG GGT TGT GG
SDF1A/CXCL12	NC_000020.11	Forward: AGG TGG TGG TGG TGG TGG TG
		Reverse: GGG GGG GTA GAA TGT GAA GG
β-actin	NC_000007.14	Forward: GGC ACC CAG CAC AAT GAA G
		Reverse: CCG ATC CAC ACG GAG TAC TTG
GAPDH	NC_000012.12	Forward: ATT GCC CCT CAA CGA CCA CTT
		Reverse: TGC TGT AGC CAA ATT CGT TGT C

### Migration assays of MSCs in response to MCP-1/CCL2 and SDF-1/CXCL12

MSCS (labeled MION-Rh) migration was performed in Transwell dishes (costar corning incorporated) 6.5 mm in diameter, with 8-μm pore filters. MSCs (4 × 10^5^/ml) in 200 μL of serum-free DMEM were added to the upper chamber and 600 μl of tested samples containing: (A) MSCs no labeled [control], (B) conditioned medium supplemented with specific neutralized antibodies anti-MCP-1/CCL2 and anti-SDF-1/CXCL12, (C) conditioned medium supplemented with specific neutralized antibodies (anti-MCP-1/CCL2), (D) conditioned medium supplemented with specific neutralized antibodies (anti-SDF-1/CXCL12), (E) CD133^+^ cell culture supernatants (TBSCM), and (F) chemokines MCP-1/CCL2 and SDF-1/CXCL12, which were placed in the lower chambers.

Recombinant MCP-1/CCL2 (MCP-1; Perprotech, NJ, USA) and recombinant human SDF-1/CXCL12 (SDF-1; R&D Systems, Wiesbaden, Germany) were diluted in serum-free DMEM to different concentrations ranging from 5 to 500 ng/ml. After overnight incubation in 5% CO_2_ at 37 °C, cells remaining on the upper face of the filters were removed with a cotton wool swab. Chambers were fixed for 20 min at room temperature with 4% formaldehyde in PBS. MSCs that had migrated through the pores and adhered to the lower surface of the membrane were analyzed under high-power (× 400) fluorescence microscopy. Each experiment was performed a minimum of three times. For migration assays, data are expressed as the mean number of cells per high-power field (cells/HPF) ± standard error (SE). Statistical analysis was performed using Student’s *t* tests. Statistical significance was set at *p* < 0.05.

### Animal ethics statement

All the experimental procedures were performed in accordance with the guidelines for animal experimentation determined by the UNIFESP Care Committee. This protocol was approved by the Committee on the Ethics of Animal Experiments of the UNIFESP. In addition, ethical conditions were maintained, assuming all international rules of animal care outlined by the International Animal Welfare Recommendations and in accordance with local institutional animal welfare guidelines.

### Tumorigenesis study through MSC action

The animals (*n* = 15; male Wistar rats) were treated with immunosuppressant drugs, anesthetized with ketamine (55 mg/kg) and treated with xylazine (11 mg/kg) for stereotaxic implantation of the cells in different conditions: (A) 1 × 10^4^ MSCs labeled MION-Rh, (B) 1 × 10^4^ CD133^+^ GBM cells labeled Qdots (705 nm), (C) 1 × 10^4^ MSCs labeled MION-Rh added 1 × 10^4^ CD133^+^ GBM cells labeled Qdots(705 nm), and (D) implantation of 1 × 10^4^ CD133^+^ GBM cells labeled Qdots (705 nm); 28 days is expected for the establishment of the GBM and infusion by caudal vein 1 × 10^4^ MSCs (MION-Rh); follow the development of tumor by 20 days.

The hair was then removed from the top of the head. The animal was subsequently fixed to the stereotaxic apparatus (Stoelting®, model 51700) using in-ear and upper teeth bars. After making a skin incision on the dorsal region of the skull and removing the periosteum, a trepanation of the bone cap was performed using a dental drill. The implantation position was determined and marked on the bone according to Swanson’s Stereotaxic Atlas guidelines at the following coordinates: 6.0 mm anteroposterior, 4.5 mm mediolateral, and a depth of 2.2 mm according by Pavon et al. [34, 35]. A Hamilton syringe was used to implant of different cells in 10 μL of culture medium into the right caudate putamen (CPu). The cells were slowly injected over a 10-min period. The syringe was kept in position for an additional 2 min before being withdrawn. The syringe was slowly raised until it was completely removed from the brain in order to avoid drawing the injected solution back into the needle. The bone was then reassembled using bone wax, and the skin sutured using cotton thread. Tumor development was monitored for 28 days and was reproducible in the five GBM samples. For the in vivo migration assay, the brain samples were collected 20 days later for cryosectioning (16-μm-thick sections) and counter staining.

### In vivo tumor development analysis by molecular imaging

Tumor development was monitored using an in vivo imaging device, Bruker model MSFXPRO. Throughout image acquisition, animals were placed in dorsal recumbency and remained anesthetized with inhaled 2% isoflurane in oxygen at 2 L/min. Initially, the skull images were acquired by X-ray. The fluorescence of the labeled cells was evaluated using the excitation (540 nm) and emission (585 nm) of MION-Rh and excitation (405–665 nm) and emission (705 nm) of Qdots (705 nm). The images were acquired and evaluated using multiplex location software.

### Magnetic resonance imaging (MRI) tumor analysis

MRI brain scans were obtained in a 2 Tesla/30 cm horizontal superconducting magnet 85310HR (Oxford Instruments, Abingdon, UK) interfaced to a Bruker Avance AVIII console (Bruker-Biospin, Ettlingen, GE) with Paravision 5.1 software (Bruker, Ettlingen, GE). A crossed saddle radiofrequency coil [37] was used as a head probe in animals anesthetized with ketamine/xylazine (95/12 mg/kg, i.p.). A T_2_-weighted RARE (Rapid Acquisition with Refocused Echoes) sequence (TR = 5000 ms, TE = 40.5 ms, RARE factor = 8, 4 averages, 6 min/animal) was used in a volume of 32 × 32 × 24 mm^3^ covered by a 128 × 128 matrix and 2-mm slice thickness without gaps (12 slices), generating a spatial resolution of 250 × 250 mm^2^. Immediately after RARE acquisition, a T_2_*-weighted image, using a FLASH (Fast Low Angle Shot) sequence (TR = 500 ms, TE = 15 ms, flip angle = 30°, 8 averages, 6 min/animal) was acquired. For this image, a volume of 32 × 32 × 24 mm^3^ was covered by a 192 × 192 matrix and 2-mm slice thickness without gaps (12 slices), generating a spatial resolution of 167 × 167 mm^2^.

### Histopathological analysis of tumor tissues

After image acquisition, the animals were anesthetized and transcardially perfused with a buffered saline solution and 4% paraformaldehyde (PFA). The brains were removed and stored in PFA for 24 h and cryoprotected in a 40% sucrose solution for 48 h. Coronal sections were cut to 40 μm in thickness using a cryostat (Leica) and stained using standard procedures for hematoxylin-eosin and Prussian Blue staining for MION-Rh and for immunohistochemical (IHC) staining for glial fibrillary acidic protein (GFAP), vascular endothelial growth factor (VEGF), proliferation marker Ki67, p53 nuclear staining, MSCs surface markers (CD44 and CD73), and CD9 exosome marker.

## Results

### The establishment of tumor subspheres of CD133^+^ selected cells from primary cell cultures of GBM

**Fig. 1:**
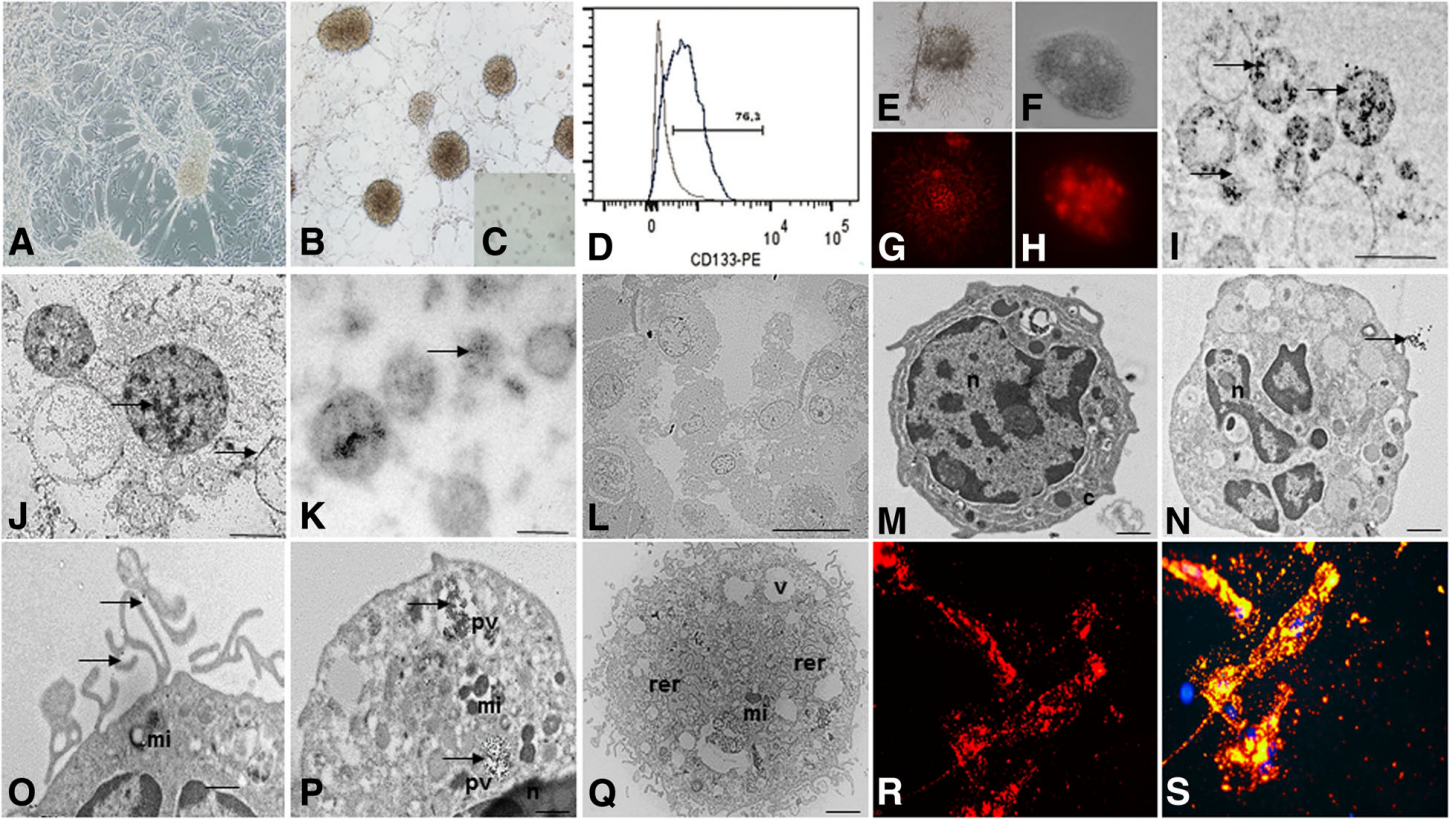
**a** The establishment of human GBM primary cell culture. **b** Isolation of tumor neurospheres derived from GBM primary cell culture. **d** Purification of GBM cells from tumor subspheres using CD133 microbeads. Immunophenotypic characterization by using flow cytometry to evaluate the efficiency of magnetic cell separation for the antigenic marker, CD133 (76.3%). **e**–**h** CD133^+^GBM cells were able to further generate subspheres, compared with the absence of subspheres obtained from CD133^−^ fractions (**c**). **e**, **f** GBM subspheres visualized by inverted microscopy. **g**, **h** GBM subspheres visualized by fluorescence microscopy. **i**–**k** TEM of the GBM subspheres. **l**–**q** TEM of the CD133^+^ stem cells. n = nucleus, c = cytoplasm, mi = mitochondria, rer = rough endoplasmic reticulum, pv = pinocytic vesicles, v = vacuoles, arrow = electron-dense granules or magnetic beads. Scale: **i**–**k** 5.0 μm, **l** 2.0 μm, **m**–**q** 1.0 μm. **r** Fluorescence detection of Qdots (705 nm) labeling in the CD133^+^ GBM cells. **s** Fluorescence detection of Qdots (705 nm) labeling in the CD133^+^ GBM cells and DAPI. Magnification: ×400. All figures are representative ones from assays performed at least five times

Primary cell cultures were successfully obtained from all GBM collected samples (*n* = 5) (Fig. 1a). These cells were homogenous, displayed fusiform format and were arranged in multidirectional bundles in culture (Fig. 1a). GBM neurospheres selected by using a CD133^+^ affinity column were able to further generate robust subspheres with well-defined morphology (Fig. 1b, h, i), whereas the negative fraction (the CD133^−^ cells) was unable to generate subspheres (Fig. 1c). The establishment of tumor subspheres of CD133^+^ selected cells was reproducible in the five GBM samples.

### Immunophenotyping of the CD133^+^ GBM cells

We obtained attached GBM populations from all five (*n* = 5) collected and processed GBM samples GBM subspheres selected by using a CD133^+^ affinity column showed a higher content (more than 70% in the five samples) of CD133 positive cells (76.3%) (Fig. 1g), which also were visualized by immunofluorescence assay (Fig. 1j, k).

### Ultrastructural characterization of GBM subspheres and CD133^+^ cells

Using electron microscopy for ultrastructural analysis, we observed the presence of electron-dense granules inside the GBM subspheres (Fig. 1d–f) and pinocytic vesicles (Fig. 1s) and also on the cell surface of the CD133^+^ cells (Fig. 1n, q), demonstrating the presence of anti-CD133 monoclonal antibodies bound to magnetic beads.

Electron micrographs also showed that the CD133^+^ cells had a round morphology (Fig. 1m, n, q, s), with some discrete cytoplasmic projections (Fig. 1q). The nuclei, with visible nucleoli, occupied majority large part of the cells (Fig. 1m). In the cytoplasm of the CD133^+^ cells, we observed the presence of circular mitochondria (Fig. 1o, p, q), rough endoplasmatic reticulum (Fig. 1q), and pinocytic vesicle (Fig. 1p).

### Detection of Qdots (705 nm) in the CD133^+^ GBM cells and MION-Rh in MSCs

**Fig. 2:**
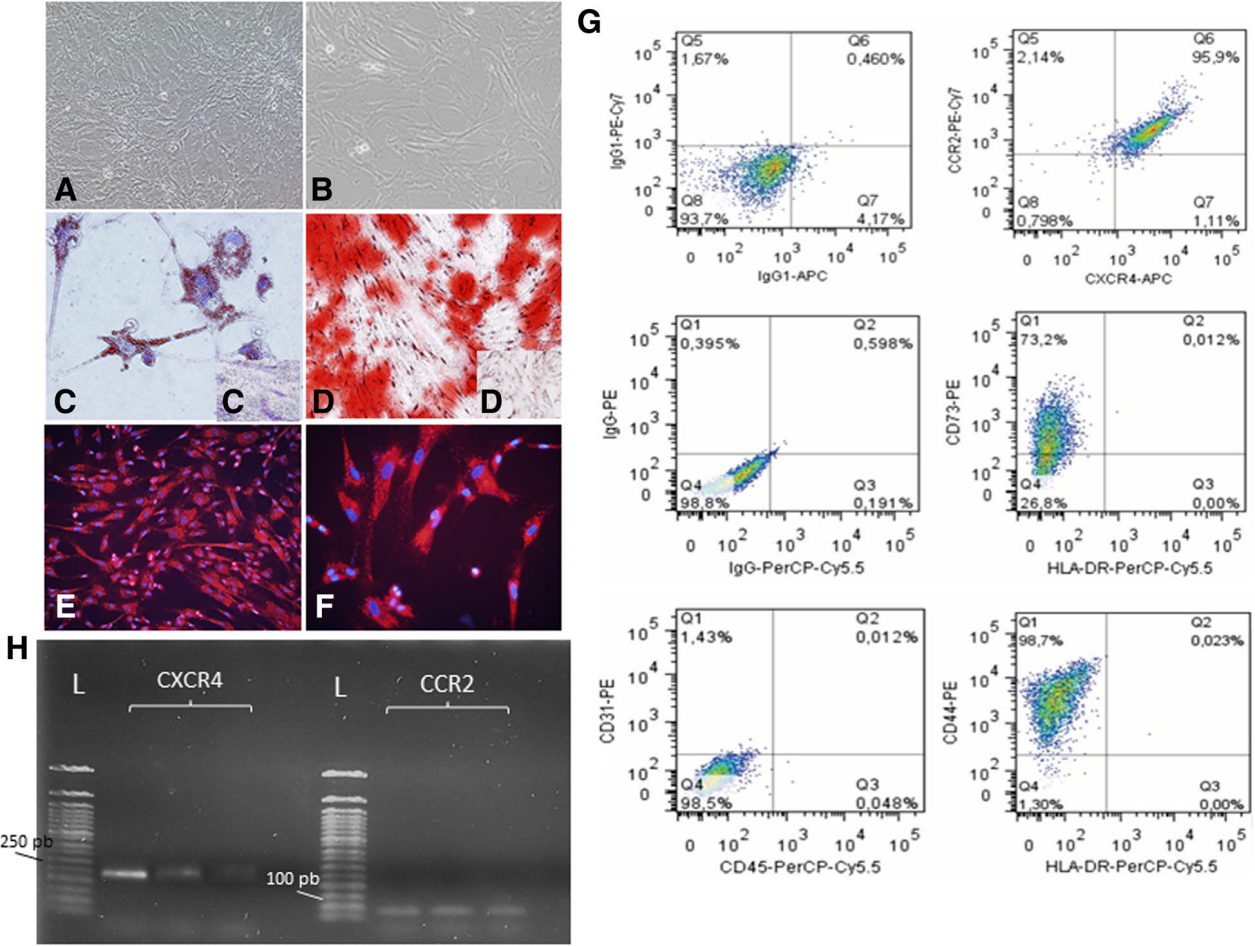
**a**, **b** Culture of MSCs with approximately 80–90% confluence. Magnification: ×100. **c** Induction of adipocyte-like phenotype by oil red stain. **d** Induction of osteogenic-like by Alizarin red stain. **c**, **d′** Undifferentiated control for adipogenic and osteogenic differentiation, respectively. **e**, **f** Fluorescence detection of MION-Rh labeling in the MSCs. Magnification: **a**, **b**, **d**, **e** ×100; **c**, **f** ×400. **g** Graphs summarize FACS analysis of MSC expression of cell markers: 73.2% of MSCs reacted with the anti-CD73 antibody; 98.7% of MSCs reacted with the anti-CD44 antibody; 95.9% of MSCs reacted with the anti-CXCR4 and anti-CCR2 antibodies and there was low or no expression of CD31 and CD45 markers. H RT-PCR analysis of CXCR4 and CCR2 mRNA levels expressed in MSCs (triplicate samples). FACS and RT-PCR analysis are representative of all collected MSCs samples

A qualitative evaluation of the intracellular distribution of Qdots (Fig. 1r, s) and MION-Rh (Fig. 2e, f) was performed by using fluorescence microscopy. The fluorescence spectrum showed that both nanoparticles were internalized as intracellular granules well distributed throughout the cytoplasm, demonstrating an effective in vitro cellular labeling.

### Isolation, culture of MSCs

After three passages of culture, the MSC population from UC samples became more morphologically homogeneous. These cell populations mostly exhibited a fibroblast-like cell profile (Fig. 2a, b). The process of differentiation of MSCS into adipocyte-like was demonstrated by the oil red cytochemical test, which exhibited, in red, lipid droplets (Fig. 2c); the differentiation of MSCs into osteoblast-like cells was also confirmed, showing a strong cytochemical pattern of Alizarin Red, which indicated the presence of calcium deposits (Fig. 2d). Thus, we confirmed that the cultured cells demonstrated multipotentiality, by giving rise to osteoblasts and adipocytes when exposed to adequate differentiating conditions. FACS analysis showed the cells were strongly positive for the typical mesenchymal markers, such as CD29, CD44 (hyaluronic receptor), CD73, CD90, CD105 (endoglin), CD166, low or no expression of MHC class I antigens, HLA-DR and hematopoietic cell markers (CD14, CD31, CD34, CD45 and CD106), and absence of MHC class II antigens (29) (Fig. 2g).

### MSCs express the chemokine receptors CCR2 and CXCR4

Before performing specific studies, we verified that the cultured cells were negative for CD31 and CD45 surface markers and positive for CD44 and CD73 surface markers (Fig. 2g).To study the role of chemokine receptors in MSC migration toward CD133^+^ GBM cells, we examined the expression of homing markers (the receptors for MCP-1/CCL2 and SDF-1/CXCL12, respectively) in MSCs, which co-expressed CXCR4 and CCR2 (95.9%) (Fig. 2g) by FACS analysis. To confirm this data, we identified the transcription of CXCR4 and CCR2 mRNAs (Fig. 2h) of MSCs by RT-PCR analysis.

### CD133^+^ GBM cells express MCP-1/CCL2 and SDF-1/CXCL12

**Fig. 3:**
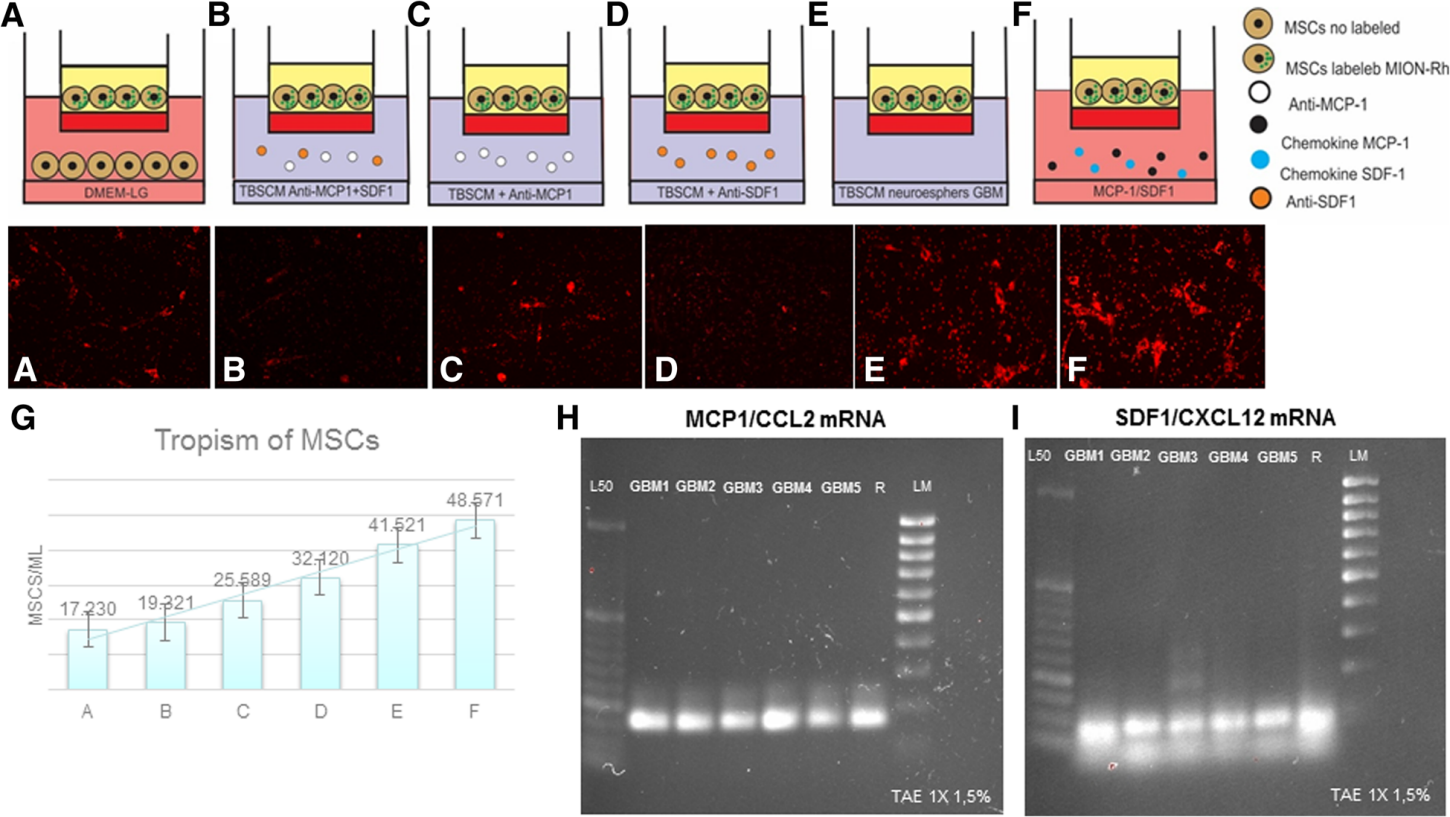
Representative figure of migration assays of MSCs, in transwell dishes, in different conditions placed in the lower chambers: **a** MSCs not labeled [control], **b** conditioned medium supplemented with specific neutralized antibodies (anti-MCP-1 and anti-SDF-1), **c** conditioned medium supplemented with specific neutralized antibodies (anti-MCP-1), **d** conditioned medium supplemented with specific neutralized antibodies (anti-SDF-1), **e** CD133^+^ cell culture supernatants (TBSCM), f chemokines MCP-1 and SDF-1, **g** graph summarized of mean number of migrated MSCs in relation to different conditions, **h** RT-PCR analysis of MCP-1/CCL2 mRNA levels expressed in CD133^+^ GBM cells (*n* = 5; GBM1 GBM2 GBM3 GBM4 GBM5). **i** RT-PCR analysis of SDF-1/CXCL12 mRNA levels expressed in CD133^+^ GBM cells (*n* = 5; GBM1 GBM2 GBM3 GBM4 GBM5). L50: Ladder 50 bp; LM low mass ladder; R positive control (human reference total RNA Clontech)

We postulated that these chemokines, released by CD133^+^ cells, could be potential mediators of MSC migration. To test this hypothesis, we examined their expression using RT-PCR. We observed that CD133^+^ GBM cells express the transcripts for MCP-1/CCL2 mRNAs (Fig. 3h) and SDF-1/CXCL12 mRNAs (Fig. 3i).

### MSCs migrate in response to MCP-1/CCL2 and SDF-1/CXCL12

To determine whether CD133^+^ GBM cells secreted chemokines that contribute to MSC chemotaxis, we incubated these cells, labeled MION-Rh, in response to the TBSCM supernatant of GBM neurospheres. In this case, we found a significant increase in MSCs (Fig. 3e) in relation to the control group (only MSCs in DMEM-LG) (Fig. 3a). Interestingly, higher concentrations of MCP-1/CCL2 and SDF-1/CXCL12 in DMEM-LG-induced migration of MSCs (Fig. 3f). We also incubated CD133^+^ GBM cells in TBSCM with anti-MCP-1/CCL2 antibody (Fig. 3c) and anti-SDF-1/CXCL12 antibody (Fig. 3d) (10 μg/ml). Addition of the anti-MCP-1/CCL2 more anti-SDF-1/CXCL12 neutralizing antibody significantly attenuated the migration of MSCs (Fig. 3b). These results were summarized in a graph (Fig. 3g), which described the mean number of migrated MSCs in relation to different conditions. This suggests that MCP-1/CCL2 and SDF-1/CXCL12 mediate MSC migration toward CD133^+^ cells.

### In vivo GBM detection by imaging and histopathological analysis

**Fig. 4:**
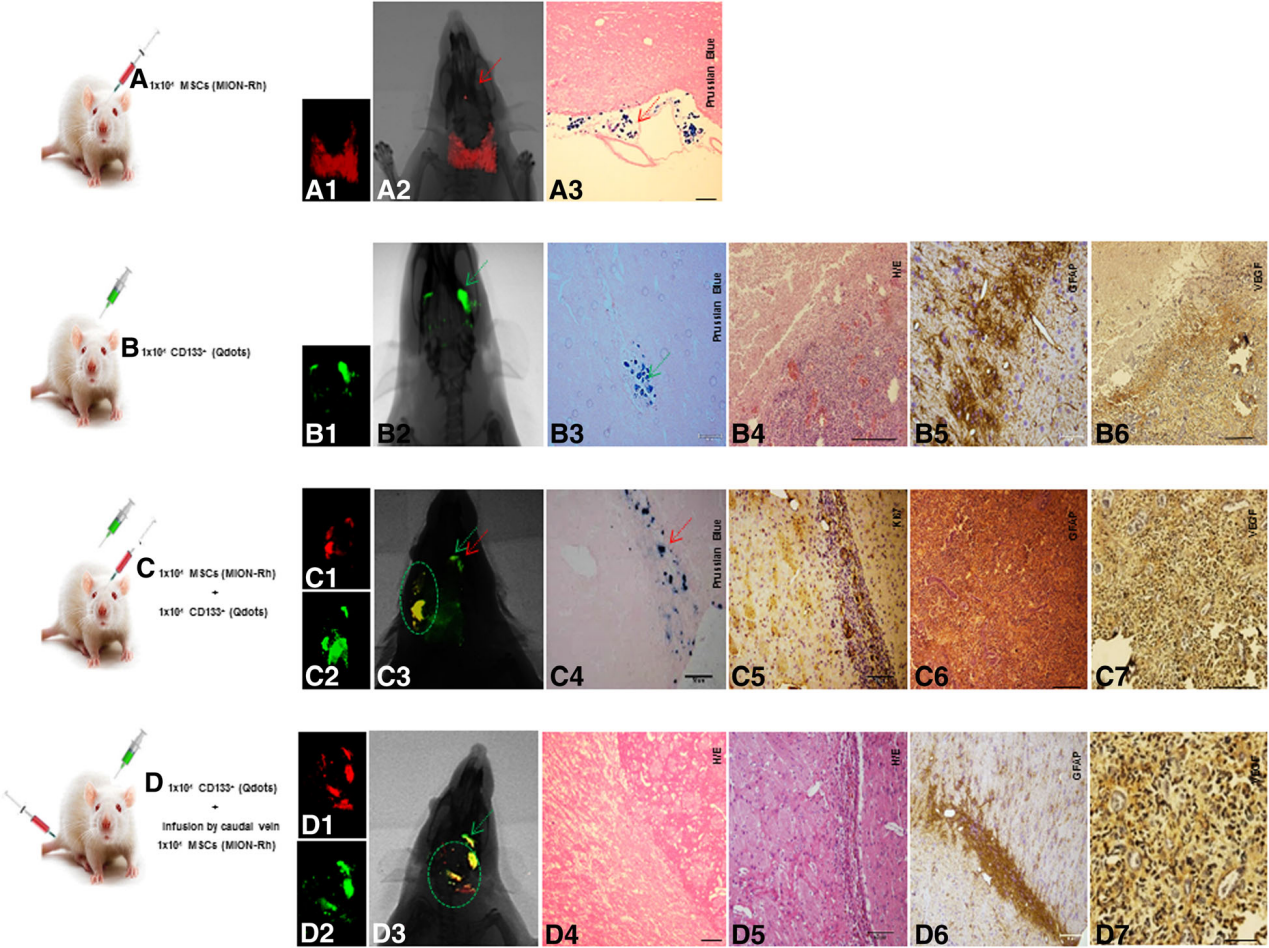
Tumorigenesis study for stereotaxic implantation of the cells in different conditions: **A** 1 × 10^4^ MSCs labeled MION-Rh; **B** 1 × 10^4^ CD133^+^ GBM cells labeled Qdots(705 nm); **C** 1 × 10^4^ MSCs labeled MION-Rh added 1 × 10^4^ CD133^+^ GBM cells labeled Qdots(705 nm); **D** implantation of 1 × 10^4^ CD133^+^ GBM cells labeled Qdots (705 nm), after the establishment of the GBM (28 days), was made the infusion in caudal vein 1 × 10^4^ MSCs (MION-Rh); the development of tumor was followed for 20 days. **A**_**1**_, **C**_**1**_, **D**_**1**_ MSCs labeled MION-Rh and visualized by fluorescence detection. **B**_**1**_, **C**_**2**_, **D**_**2**_ CD133^+^ GBM cells labeled Qdot 705 nm and visualized by fluorescence detection. **A**_**2**_, **B**_**2**_, **C**_**3**_, **D**_**3**_ MSCs labeled MION-Rh and CD133^+^ GBM cells labeled Qdot 705 nm using combined fluorescence and X-ray detection. **A**_**3**_, **C**_**4**_ IHC analysis for Prussian blue staining of the MSCs labeled with MION-Rh. **B**_**4**_, **D**_**4**_, **D**_**5**_ Hematoxylin and eosin staining. **B**_**5**_, **C**_**6**_, **D**_**6**_ IHC analysis for GFAP. **C**_**5**_ IHC analysis for Ki67; **B**_**6**_, **C**_**7**_, **D**_**7**_ IHC analysis for VEGF. Red arrow: site of stereotaxic implantation of MSCs labeled MION-Rh. Green arrow: site of stereotaxic implantation of CD133^+^ GBM cells labeled Qdot (705 nm).Green circle evidenced proliferation: MSCs and CD133^+^ GBM cells. These images are representative of all collected MSCs and GBM samples

The progression of tumor growth, generated after stereotaxic implantation of CD133^+^ GBM cells labeled with Qdots (705 nm) (Fig. 4B, representative image of the whole group) was clearly detectable using combined fluorescence and X-ray detection (Fig. 4B_1, 2_) on day 28. Histopathological examination showed that the tumors exhibited high cellularity, nuclear atypia, and invasiveness (Fig. 4B_4_). Immunohistochemical analysis for GFAP confirmed tumor formation originated in the glia (Fig. 4B_5_) and vascular proliferation (Fig. 4B_6_).

### Tumorigenesis and MSCs

The in vivo study began with the exclusive stereotaxic implantation of 1 × 10^4^ MSCs labeled MION-Rh (condition A), which, as expected, was not capable of generate tumor. These cells were visualized outside the brain parenchyma surface by using combined fluorescence and X-ray detection (Fig. 4A_1, 2_) and by the Prussian Blue histochemical assay, which demonstrated the presence of iron (MION-Rh) (Fig. 4A_3_). This analysis was used as the control of the study (these cells alone did not generate brain tumor).

The progression of tumor growth, generated after stereotaxic implantation of 1 × 10^4^ CD133^+^ GBM cells labeled with Qdots (705 nm) (condition B) was showed in Fig. 4b. The tumor was identified by using combined fluorescence and X-ray detection (Fig. 4B_1, 2_) and by the Prussian Blue histochemical analysis (Fig. 4B_3_). This analysis served as control group in the process of comparison of tumor development of the C and D conditions.

Interestingly, when 1 × 10^4^ MSCs labeled MION-Rh were implanted together with 1 × 10^4^ CD133^+^ GBM cells labeled Qdot 750 nm (condition C), the tumor displayed significant progression on the contralateral side, in which also was evidenced migration of MSCs labeled MION-Rh (Fig. 4C_4_; representative image of the whole group). The tumor was identified by using combined fluorescence and X-ray detection (Fig. 4C_3_; representative image of the whole group) and by IHC analysis, which demonstrated significant aggressiveness, glial invasiveness (Fig. 4C_6_; representative image of the whole group), vascular proliferation (Fig. 4C_7_) and the detection of a high number of cycling cells (Fig. 4C_5_; representative image of the whole group).

**Fig. 5:**
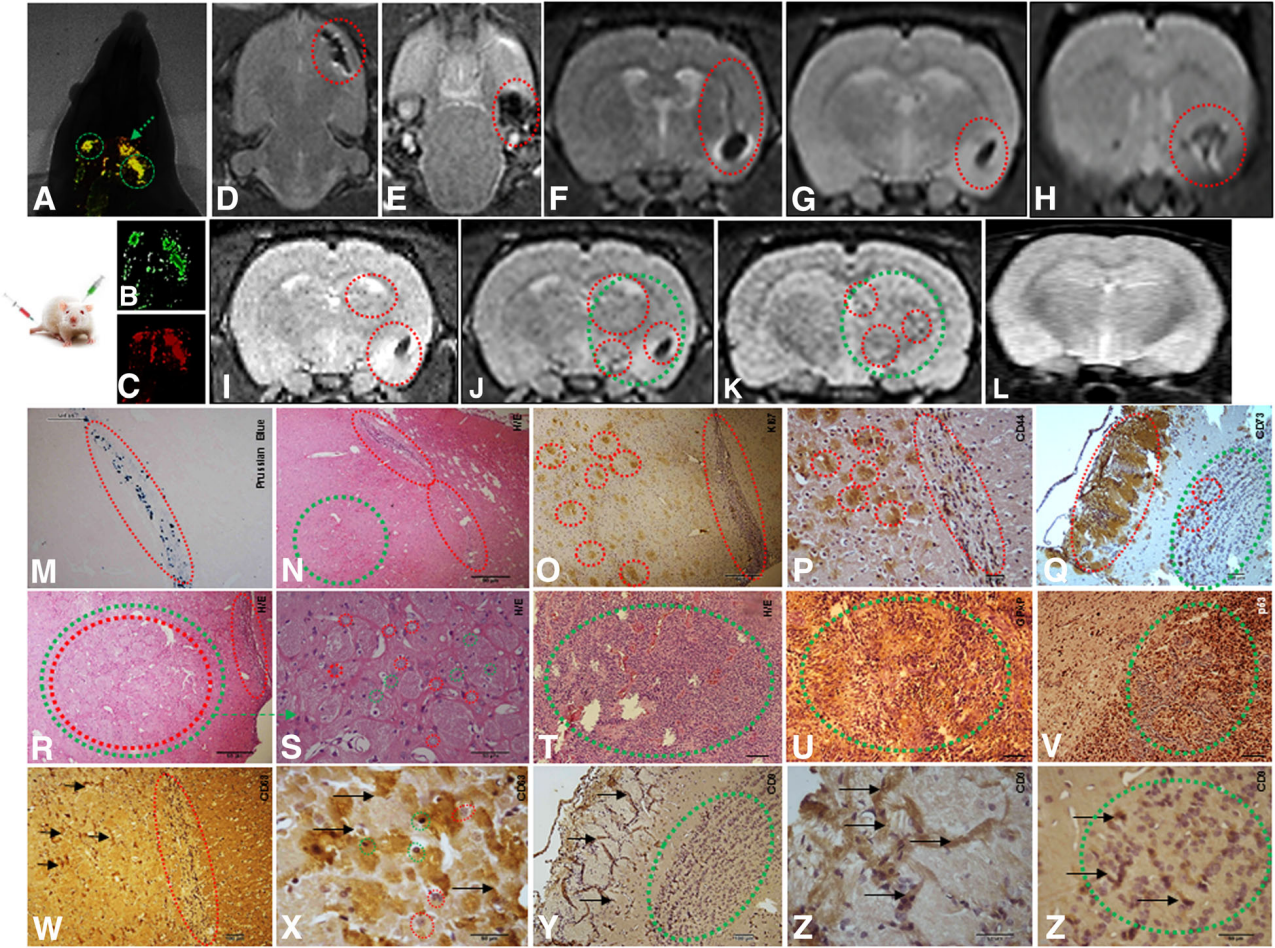
Implantation of 1 × 10^4^ CD133^+^ GBM cells labeled Qdots (705 nm), after the establishment of the GBM (28 days), was made the infusion in caudal vein 1 × 10^4^ MSCs (MION-Rh); the development of tumor was followed for 20 days. **a** MSCs labeled MION-Rh and CD133^+^ GBM cells labeled Qdot 705 nm using combined fluorescence and X-ray detection. **b** CD133^+^ GBM cells labeled Qdot 705 nm and visualized by fluorescence detection. **c** MSCs labeled MION-Rh and visualized by fluorescence detection. **d**–**h**, **i**–**l** MRI (T_2_*-weighted images) of animal brain monitoring of the process of migration of MSCs, which were able to cross the blood-brain barrier of the animal and migrated to the tumor region, promoting GBM cell proliferation. **l** MRI (T_2_*-weighted images) of animal brain without stereotaxic implantation of cells (control group). Red circle showed migration assays of MSCs and green circle evidenced tumor propagation. **m** IHC analysis for Prussian blue staining of the MSCs labeled with MION-Rh. **n**, **r**, **s**, **t** Hematoxylin and eosin staining. **u** IHC analysis for GFAP. **o** IHC analysis for Ki67; **v** IHC analysis for p53; **p** IHC analysis for CD44 staining of the MSCs; **q** IHC analysis for CD73 staining of the MSCs; **w**, **x** IHC analysis for CD63 staining of the MSCs-derived exosomes; **y**–**z**′ IHC analysis for CD9 staining of the MSCs-derived exosomes. These images are representative of all collected MSCs and GBM samples

To determine the contribution to MSC chemotaxis of chemokine-secreting CD133^+^ GBM cells, we injected 1 × 10^4^MSCs into the caudal vein of the animals, after the tumor had been established for 28 days (condition D) (Figs. 4D and 5; representative image of the whole group). Corroborating the results of in vitro migration assays, MSCs labeling MION-Rh were able to cross the blood-brain barrier (Figs. 4D_1_ and 5c, o), co-locating CD133^+^ cells (Figs. 4D_3_, 5a) and promoting their proliferation (Figure 4D_2_ and Fig. 5b), when we follow the development of the tumor for 20 days.

IHC analysis confirmed tumor dissemination (Figs. 4D_4, 5_ and 5n, q–t), glial invasiveness (Figs. 4D_6_, 5u), vascular proliferation (Fig. 4D_7_), and IHC staining patters of p53 (Fig. 5v), when compared to the study situation that did not receive MSCs.

This study condition was also monitored using MRI analysis (Fig. 5; representative image of the whole group) to evaluate migration of MSCs labeled with MION-Rh, a nanoparticle suitable for the study, due to its magnetic character, in addition to fluorescence. We observed “dark” hypointense zones in the T_2_*-weighted images (Fig. 5d–k), which revealed the process of migration of MSCs toward CD133^+^ GBM cells, this outcome also was visualized by presence of iron (MION-Rh) in the histochemical analysis (Fig. 5m). MSCs also were visualizes by cell proliferation assay (Fig. 5o and expression of CD44 (Fig. 5p) and CD73 (Fig. 5q) positive cell surface markers by IHC. These regions were also, respectively, positives for CD63 (Fig. 5w, x) and CD9 (Fig. 5y, z, z′) exosomes markers, which, probably, were secreted by MSCs (Fig. 5q, r, y, z).

## Discussion

The tropism of MSCs toward GBM makes these cells as a highly attractive vehicle for the delivery of therapeutic products directly to tumor. Our results demonstrated that possibly this tropism should be governed by CD133^+^ GBM cells. However, the molecular events that govern MSCs homing to CD133^+^ GBM cells and their effects on tumor development are unclear.

Herein, we isolated CD133^+^ GBM cells, which were appropriately obtained from the establishment of tumor subspheres from primary cell cultures of GBM and characterized by immunophenotype and ultrastructural aspects described elsewhere [33, 34].

In our study, we demonstrated that specific chemokines, such as MCP-1/CCL2 and SDF-1/CXCL12 (receptors CCR2 and CXCR4 expression in MSCs from hUCB), mediate the migration of MSCs toward CD133^+^ GBM cells in vitro.

MCP-1/CCL2 is a member of the cytokine/chemokine superfamily that regulates migration and infiltration of monocytes/macrophages to tumor sites, [38, 39], thereby inhibiting anti-tumor immune responses [38] and promoting tumorigenesis and metastasis of the gliomas in vivo [40]. Moreover, addition of the anti-MCP-1/CCL2 neutralizing antibody significantly attenuated the migration of MSCs toward CD133^+^ cell culture supernatants (TBSCM). The chemokine MCP-1/CCL2 plays an important role in the regulation of stem cell trafficking [41]. Recent studies demonstrate that the progress of GBM is driven by stem cells, critical promoters of tumor growth, invasion, and neovascularization [42]. CXCR4 has been found to be upregulated in CD133^+^ GBM stem cells upon activation with SDF-1/CXCL12, a CXCR4 ligand [43]. There is evidence that disruption of CXCR4 results in a reduction of GBM stem cell markers and reduction in tumor cell proliferation [44]. Therefore, in our results in vitro, MCP-1/CCL2 and SDF-1/CXCL12 might play an important role in MSCs homing toward CD133^+^ cells.

Park et al. [45] already stated that SDF-1(CXCL12)/CXCR4 could be involved in the recruitment of MSCs to U-251MG glioma cells lines and that overexpression of CXCR4 might be a useful tool for stem cell-based glioma therapy. These authors performed the animal model using migration assay by injection of MSCs directly in the brain, the contralateral site of gliomas, and did not observe tumor dissemination after 10 days post-injection.

Considering the relevance of the CD133^+^ model [5–7], our group used this methodology to generate tumor in vivo*.* Different from Park et al. [45], we infused MSCs in the caudal vein of the animals, which were able to cross the blood-brain barrier and co-located with CD133^+^ GBM initiating cells, obtained from tumor subspheres from primary cell cultures of GBM. Following the migration protocols for 20 days, we validated the chemotactic effect of MCP-1/CCL2 and SDF-1/CXCL12 in mediating the migration of MSCs toward CD133^+^ GBM cells, and we observed tumor development, glial invasiveness, vascular proliferation and detection of a high number of cycling cells, when compared to the study situation that did not receive MSCs. MRI analysis confirmed the process of migration of MSCs toward CD133^+^ GBM cells and intense brain tumor dissemination. These findings assume that chemokines mediate MSC migration toward CD133^+^ GBM cells and that this could promote tumor development and metastatic proliferation.

Interestingly, in the study conditions, where MSCs were implanted together with CD133^+^ GBM cells, significant tumor progression was also displayed when compared to condition B, which was generated by implantation of CD133^+^ GBM cells only.

Pavon et al. [33] showed that CD133^+^ GBM cells express molecular signatures of MSCs. Therefore, we hypothesize that CD133^+^ cells, due to their MSC-like properties, recruit MSCs, and sustain tumor growth, which is affected by the tumor microenvironment created by the non-neoplastic stroma composed of inflammatory [34, 46]. MSCs release many promigratory chemokines, which facilitate tumor progression including proliferation, senescence, angiogenesis, epithelial mesenchymal transition, immune evasion, and metastasis [47, 48].

**Fig. 6:**
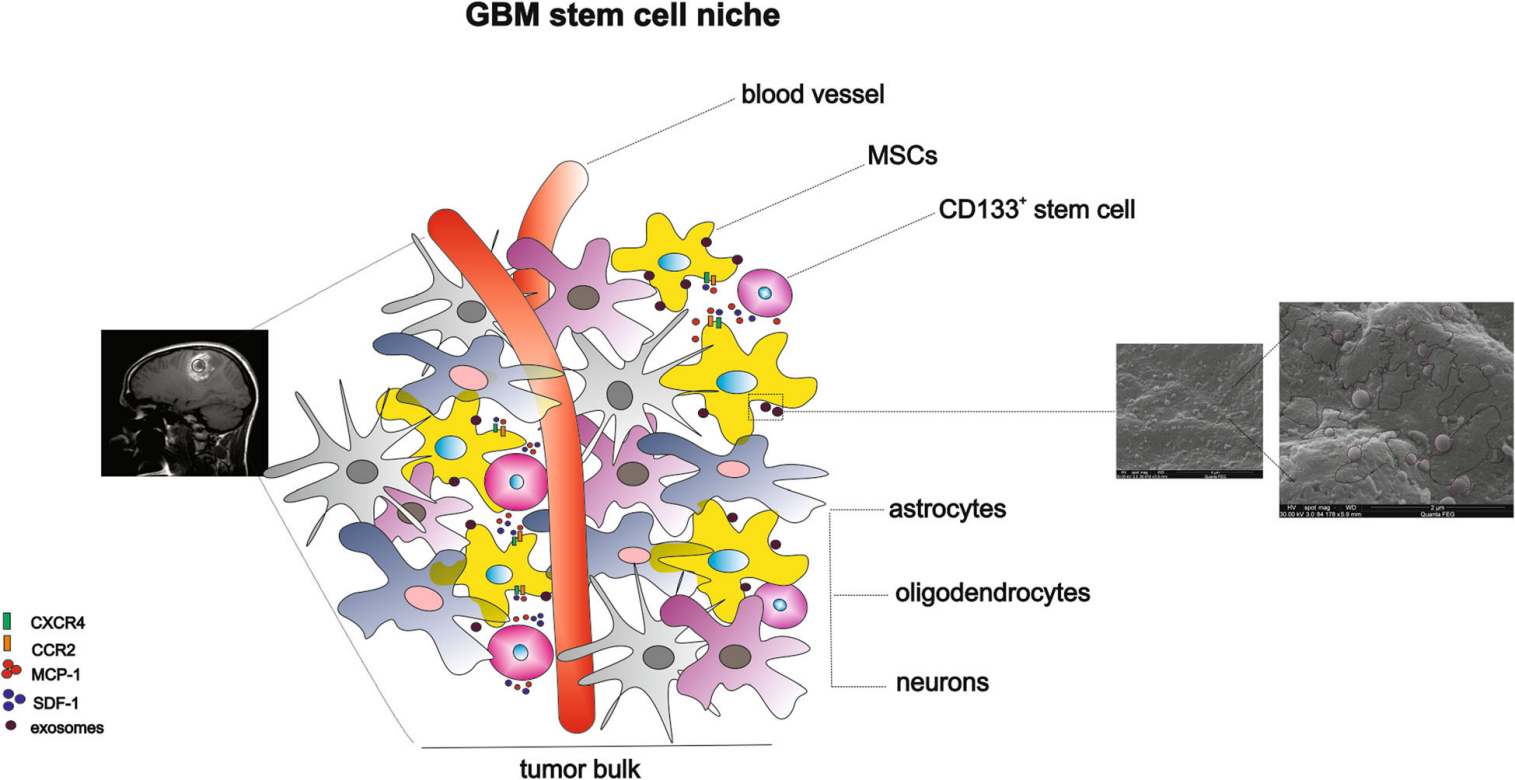
Schematic representation demonstrating that chemokines mediate MSC migration toward CD133^+^ stem cell of GBM and scanning electron microscopy of exosome, secreted by MSCs, promoting tumor dissemination

These events could be modulated by recruited MSCs-derived exosome, here in our study demonstrated by expression tetraspanin CD9/CD63 protein [49], which apparently could be involved in tumor cell invasion and consequently tumor dissemination (schematic representation described of Fig. 6). However, other studies on biological effects mediated by these vesicles need to be developed to prove this finding.

Therefore, tumor growth effect of MSCs tropism toward GBM remains controversial: (i) CD133^+^ GBM cells maintain only a subset of primary GBM; probably, CD133^−^ cells also participate in the process of modulating the tropism and (ii) the intrinsic factor such as dose of MSCs and timing of implantation should be tested in future trials.

Different studies reported either MSC anti-tumor activity or their support to tumor growth. Behaan et al. [50] and Motaln and Turnsek [51] demonstrated that the using of MSCs as cellular vectors for modulating cytokines and cytokine receptors’ signaling in GBM could been more efficient at inhibiting GBM progression. Nevertheless, it is still controversial whether this tropism of MSCs toward the tumor area is associated with GBM promotion or suppression [52]. Okamoto et al. [53] indicated that MSCs were capable of stimulating GBM cell proliferation through a paracrine effect mediated by TGFB1. These findings provide novel insights to better understand the relationship between CD133^+^ GBM cells and MSCs, raising awareness in the use of MSCs as therapies for gliomas [54]. These studies, however, explored insufficient in vivo results. In our work, we demonstrated of tumorigenicity of CD133^+^ cells in conjunction to with the migration of MSCs toward GBM and suggested, strongly, MSCs contribution to tumor development, invasion, and metastatic dissemination.

## Conclusion

We suggest that the MSC-like properties of CD133^+^ GBM cells confer proangiogenic and anti-apoptotic characteristics that may sustain tumor growth. Thus, tumor progression may be directed by reciprocal interaction between stromal cells and tumor cells, by chemotactic action of MCP-1/CCL2 and SDF-1/CXCL12 and probable, effect of MSC-derived exosome, to create an appropriate environment for tumor aggressiveness. These findings may be hardly evaluated in any future in treatment strategies that use MSCs as vehicles for drug delivery into glioma tumors.
